# Decoupled genomic elements and the evolution of partner quality in nitrogen‐fixing rhizobia

**DOI:** 10.1002/ece3.1953

**Published:** 2016-01-28

**Authors:** Benjamin R. Gordon, Christie R. Klinger, Dylan J. Weese, Jennifer A. Lau, Patricia V. Burke, Bryn T. M. Dentinger, Katy D. Heath

**Affiliations:** ^1^ Department of Plant BiologyUniversity of Illinois Urbana‐Champaign505 S. Goodwin Ave.UrbanaIllinois61801; ^2^ Department of BiologySt. Ambrose University518 West Locust StDavenportIowa52803; ^3^ Kellogg Biological Station and Department of Plant BiologyMichigan State University3700 E. Gull Lake DriveHickory CornersMichigan49060; ^4^ Jodrell LaboratoryRoyal Botanic GardensKewRichmondSurreyTW9 3DSUnited Kingdom

**Keywords:** Cheating, mutualism, nitrogen deposition, partner quality, Rhizobium, symbiosis

## Abstract

Understanding how mutualisms evolve in response to a changing environment will be critical for predicting the long‐term impacts of global changes, such as increased N (nitrogen) deposition. Bacterial mutualists in particular might evolve quickly, thanks to short generation times and the potential for independent evolution of plasmids through recombination and/or HGT (horizontal gene transfer). In a previous work using the legume/rhizobia mutualism, we demonstrated that long‐term nitrogen fertilization caused the evolution of less‐mutualistic rhizobia. Here, we use our 63 previously isolated rhizobium strains in comparative phylogenetic and quantitative genetic analyses to determine the degree to which variation in partner quality is attributable to phylogenetic relationships among strains versus recent genetic changes in response to N fertilization. We find evidence of distinct evolutionary relationships between chromosomal and pSym genes, and broad similarity between pSym genes. We also find that *nifD* has a unique evolutionary history that explains much of the variation in partner quality, and suggest MoFe subunit interaction sites in the evolution of less‐mutualistic rhizobia. These results provide insight into the mechanisms behind the evolutionary response of rhizobia to long‐term N fertilization, and we discuss the implications of our results for the evolution of the mutualism.

## Introduction

N (Nitrogen) is a critical nutrient for all plants and animals, and N availability is a major determinant of ecology in terrestrial communities (Sterner and Elser 2002; Craine 2009; Grman et al. 2010). Human activities are drastically altering the N cycle via fossil fuel combustion, the Haber–Bosch process (an industrial process that reduces N_2_ to NH_4_^+^), and modern agricultural practices – all of which add millions of tons of N to terrestrial environments globally. These anthropogenic activities represent 45% of all N fixed on Earth, and they double the rate of natural terrestrial N fixation, which is primarily driven by microbial processes and supplemented by heat shock associated with lightning strikes (Canfield et al. 2010). It is estimated that up to 50% of anthropogenic N fertilizer is lost to water, the atmosphere, and uncultivated soils (Vitousek 1997; Vitousek et al. 1997; Smil 2002). The ecological effects of this N deposition are detrimental to natural communities, ranging from decreases in biodiversity (Tilman 1987; Clark and Tilman 2008) to coastal dead zones arising from eutrophication (Diaz and Rosenberg 2008). Furthermore, increases in N availability are predicted to cause the breakdown of a resource mutualism of major ecological and economic importance – that between legumes and symbiotic rhizobia (Sprent and Sutherland 1987; West et al. 2002; Kiers et al. 2006; Oono et al. 2009; Sachs et al. 2011; Frederickson 2013).

Mutualisms, cooperative partnerships between species, are both diverse and widespread, playing key roles in the evolution and ecology of land–plants, marine ecosystems, the biology of herbivores, and more (Saffo 2014). The diverse roles played by mutualisms, such as the maintenance of biodiversity and nutrient cycling (Stachowicz 2001), are widely distributed globally (Brussaard et al. 2007), and of recognized economic importance (Pimentel et al. 1997; Vitousek et al. 1997). Despite the apparent long‐term stability of many mutualistic relationships, models predict that mutualisms should be extremely susceptible to the invasion of less‐beneficial genotypes, which give less than they take from their symbiotic partners (West et al. 2007), leading to mutualism breakdown (Sachs et al. 2004; Holland and DeAngelis 2010). Such mutualism breakdown might be exacerbated under global change (Six 2009; Kiers et al. 2010). This prediction arises from a large body of work indicating that mutualisms are quite context‐dependent, making cost–benefit ratios for partner fitness, and ultimately mutualism stability, highly contingent upon the surrounding environmental conditions (Bronstein 1994; Chamberlain et al. 2014). Moreover, because partner species often have very different physiologies, phylogenetic origins, and environmental responses, the frequencies with which partner species interact might also shift in response to a rapidly changing environment and lead to mutualism breakdown (Sachs and Simms 2006).

Leguminous plants obtain N by pairing with soil bacteria called rhizobia, which are housed in root nodules and therein fix atmospheric dinitrogen (N_2_) to ammonium (NH_4_+) in exchange for plant photosynthates. Given that N is a key traded resource in this mutualistic symbiosis, theory predicts that excess N will destabilize the legume–rhizobium mutualism and lead to the evolution of less‐cooperative rhizobia through several potential selective mechanisms (Akçay and Simms 2011). While host control mechanisms that favor cooperative rhizobium partners have been shown to remain active in high N, at least in the short‐term (Kiers et al. 2006; Regus et al. 2014), long‐term studies are needed to understand how the mutualism evolves when exposed to increased N over many generations. Indeed in a recent study, Weese et al. (2015) demonstrated that populations of *Rhizobium leguminosarum* bv. *trifolii* (*Rlt)* were less beneficial for their clover host plants after long‐term exposure (22 years) to N fertilizer, and moreover that these N‐evolved versus control strains were not phylogenetically distinct (i.e., were not separate, monophyletic lineages). Nevertheless, the evolutionary processes leading to this phenotypic differentiation were not investigated.

Rhizobia are excellent models for studying the evolution and breakdown of cooperation in changing environments, and for microbial adaptation in general. Bacteria are experimentally tractable, have short generation times, and harbor relatively small genomes, simplifying the detection of evolutionary responses (Elena and Lenski 2003). Ecological shifts in taxonomic composition and/or abundance have been shown to occur within natural bacterial communities in response to changing environments (Allison and Martiny 2008; Logares et al. 2013; Newton et al. 2013; Youngblut et al. 2013). Yet despite recent investigations, we still lack fundamental knowledge on how bacterial populations in nature evolve in response to environmental changes. While adaptive variants in bacteria can arise from point mutations (Stallforth et al. 2013), bacteria can also evolve via recombination, or by HGT (horizontal gene transfer) (Gogarten et al. 2002; Ochman & Moran 2001), “the non‐genealogical transmission of genetic material from one organism to another” (Goldenfeld & Woese 2007). HGT can potentially facilitate rapid evolutionary responses to environmental stress (Jain et al. 2003; Beaber et al. 2004), or to exposure to correlated environments of increased complexity (Mozhayskiy and Tagkopoulos 2012). If recombination is infrequent compared to mutation, then the entire genome should sweep to fixation along with a newly selected locus (i.e., the “ecotype model” (Koeppel et al. 2008)); by contrast, frequent recombination in natural bacterial populations could break up the linkage between selected and other loci (i.e., similar to classical models of eukaryotic adaptive evolution). Existing data on recombination within and among genomic elements in rhizobia is mixed (Orozco‐Mosqueda et al. 2009), and moreover depends on the phylogenetic scale analyzed. Some studies have found congruent phylogenies consistent with a general lack of recombination at the species level in contrast to abundant recombination at the population level (Wernegreen et al. 1997; Kumar et al. 2015), while others have found a high level of consistency between the chromosome and symbiosis plasmids even at the population scale (Wernegreen and Riley 1999; Bailly et al. 2007; Epstein et al. 2012). Studies of other microbial species suggest that recombination is abundant (Reno et al. 2009; Cadillo‐Quiroz et al. 2012; Shapiro et al. 2012; Held et al. 2013), and that the occurrence of genomewide sweeps is rare (Polz et al. 2013).

The *Rhizobium leguminosarum* reference genome (bv. *trifolii* WSM1689*)* has a 4.85 Mb chromosome and five plasmids varying in size from 259 to 609 Kb (Genbank accession GCA_000517615.1). In rhizobia, key genes necessary for nodulation (*nod*) and N fixation (*nif* and *fix*) have been shown to cluster (Young et al. 2006), and often reside either on a symbiosis plasmid (pSym) or as part of a genomic SI (symbiosis island). In *Rlt,* they have previously been shown to localize to the pSym (López‐Guerrero et al. 2012; Ormeño‐Orrillo and Martínez‐Romero 2013). The ability to fix N in rhizobia is derived from nitrogenases, which constitute a class of complex enzymes that catalyze biological nitrogen fixation by reducing dinitrogen to ammonia. *Rlt* possess the commonly studied Mo‐dependent nitrogenase which is primarily composed of a heterotetrameric MoFe‐core encoded by *nifD* and *nifK* that catalyzes the reduction of nitrogen to ammonia, and a dinitrogenase reductase Fe‐subunit encoded by *nifH* that provides electrons to nitrogenase and ATP‐derived energy for catalysis (Stacey et al. 1992; O'Carroll and Dos Santos 2011). Previous studies have found evidence that *nifH* has a different history of HGT from the *nifDK* enzyme core and that genetic divergence of *nifH* and 16S rRNA genes does not commonly correlate well for microbial species (Gaby and Buckley 2014). Haukka et al. (1998) concluded that the phylogeny of *nifH* was generally inconsistent with 16S, but was broadly similar to that of *nod* genes. Indeed, phylogenetic trees based on sequences of *nod* genes are generally not congruent with those based on 16S rDNA sequences, but the *nod* trees show some correlation with host plant range (Wernegreen and Riley 1999).

The extent to which sequence variation accounts for recent phenotypic divergence in partner quality should differ between loci, with strong phylogenetic signal near‐causative loci; here, we expect that strains of similar partner quality will have less sequence variation near‐causative loci. Moreover, the degree to which genomewide (versus unlinked/site‐specific) sweeps contribute to phenotypic divergence should be reflected by the degree to which all loci in the genome display phylogenetic signal; for example, without recombination we would expect the phylogenetic signal near‐causative loci to be reflected throughout the genome to some degree. Here, we use the collection of N‐evolved and control *R. leguminosarum* strains previously isolated from the long‐term N deposition experiment at the Kellogg Biological Station LTER site (Weese et al. 2015) in phylogenetically informed generalized linear mixed models and comparative phylogenetic analyses to determine the congruence between strains of *Rlt* based on pSym genes and the chromosomal ITS (internal transcribed spacer) region that lies between the 16S and 23S rRNA genes, and to estimate evolutionary relationships among N‐evolved and control strains. We chose *nifD* and *nifH* as they encode key components of the nitrogenase enzyme complex, and the *nodD‐A* spacer as a marker of nodulation genes. If *nif* explains the majority of partner quality variation, this would suggest that a related functional change, such as a reduction in N fixation efficiency, has driven changes in partner quality. Alternatively, if *nod* explains the majority of partner quality variation, changes to the flavonoid‐inducible regulatory cascade that coordinates the expression of symbiotic genes with nodule development, or potentially a host range shift may have driven the decrease in partner quality. Finally, the degree to which these loci are congruent is illustrative of the relative importance of recombination and HGT on their evolution.

## Methods

### Study system and sequencing

Briefly, our study focuses on a collection of *Rhizobium leguminosaurum* bv. *trifolii* strains (from Weese et al. 2015) previously isolated from a long‐term N fertilization experiment at the Kellogg Biological Station LTER site (Huberty et al. 1998). In previous work, we performed a single‐inoculation experiment in which three species of clover were inoculated with 63 strains isolated from either N fertilized (28 strains) or control plots (35 strains). Rhizobium strains from N‐fertilized plots were of lower mutualistic partner quality (i.e., they resulted in plants with fewer leaves and biomass and lower chlorophyll content). See Weese et al. (2015) for full methods on rhizobium isolations, quantitative trait phenotyping, and sequencing of the ITS region. Each strain was grown in TY (Vincent 1970) liquid media prior to DNA extraction via the Qiagen Gentra Puregene Yeast/Bacteria kit (Qiagen, Valencia, CA). Three loci from the pSym‐located symbiosis island were amplified via PCR: *nifH* and *nifD*, which encode dinitrogenase reductase and the *α*‐subunit of dinitrogenase (which are the key components of the nitrogenase enzyme complex), respectively, and the *nodD‐A* region, which spans the intergenic region of *nodD* and *nodA* genes. PCR was performed using the GoTaq PCR protocol (Promega), and successfully amplified samples were sequenced at the University of Illinois at Urbana‐Champaign W. M. Keck Center for Sanger sequencing. All ambiguous base calls were manually confirmed using Sequencher 5.1 (Gene Codes Corporation Ann Arbor, MI). The accession number for NifD used in the protein alignment is YP_009081007 (*Rhizobium leguminosaurum* bv. trifolii) (Miller et al. 2007). All datasets were initially aligned using MUSCLE (Edgar 2004) and then trimmed of uneven ends and examined for potential nonhomologs using SeaView 4.5.3 (Gouy et al. 2010). MAFFT v7.221 (Katoh and Standley 2013) was used to realign the 55 ITS sequences from strains for which nifD genes were available with the Q‐INS‐i algorithm, and the 36 nodD sequences with the L‐INS‐i algorithm. For each gene comparison, datasets were pruned of taxa that were missing data from one of the two genes, so that all datasets consisted of identical taxa. Separate ML analyses were run for all individual datasets using the GTRGAMMA model in RAxML (Stamatakis 2014).

### Phylogenetic and mixed model analyses

Unless otherwise noted, tests of congruence and recombination were implemented using R (R Core Team 2013). We first applied the SH (Shimodaira–Hasegawa) test, a widely used nonparametric maximum likelihood test. SH tests the null hypothesis that all tested topologies are equally good explanations of the data by comparing the log likelihood values from two or more trees. Even when the number of trees compared is minimized, the SH test is known to be conservative (Buckley 2002). For each gene dataset, the best ML tree for that gene was compared with the best ML topology for the competing gene using the SH test implemented in PAUP* v4.0a146 (Swofford 1993). The likelihood scores for each tree were calculated by estimating model parameters under a GTR+G model, and the SH test was carried out using RELL bootstrapping (100,000 replicates) in PAUP*v4.0a146 (Swofford 1993). In addition to the character‐based SH test, we also applied a topology‐based test to calculate the measure of incongruence depicted by tanglegrams. Following Pérez‐Escobar et al. (2015), we applied a PACo (Procrustean Approach to Cophylogeny) analysis (Balbuena et al. 2013) implemented in the *ape* and *vegan* packages (Paradis et al. 2004; Oksanen et al. 2013) to provide a measure of topological congruence by assessing the degree to which one phylogenetic distance matrix predicts another (i.e., is significantly better than random) using a goodness‐of‐fit statistic with significance established by randomization of the phylogeny association data.

Briefly, using packages *adephylo, ape,* and *phylobase* (Paradis et al. 2004; Jombart et al. 2010; Bolker et al. 2013), we utilized *phylo4d()* (Bolker et al. 2010) to combine our phylogenies and dataset to visually assess the distribution of partner quality on the phylogenies. Finally, to visually assess whether incongruence was restricted to a particular clade or region of the tree, we formed tanglegrams using the software Dendroscope 3 (Scornavacca et al. 2011; Huson and Scornavacca 2012). The amount of tangle, or the degree to which lines cross, is a rough approximation of topological incongruence, and while tanglegrams ignore support values on the trees, they allow a determination of whether incongruence segregates by region or clade.

We used phylogenetic GLMMs (generalized linear mixed models) to estimate the proportion of rhizobium partner quality variation attributable to phylogenetic variation (i.e., linked to the phylogenetic marker locus) versus the recent N fertilization treatment (i.e., changes elsewhere in the genome in response to N fertilization). This analysis, implemented using the MCMCglmm package in R (Hadfield and Nakagawa 2010), jointly estimates the effects of a phylogenetic distance matrix and other variables on quantitative trait variation (chlorophyll content, leaf number, root mass, shoot mass, and stolon number), similar to the “animal model” of quantitative genetics (Wilson et al. 2010). We ran separate models that included N treatment and either the ITS phylogeny or the pSym phylogenies. We followed the recommendations specified by Hadfield (2010) and Villemereuil et al. (2012) for stipulating the MCMCglmm priors by dividing the observed phenotypic variance evenly between the effects (the phylogeny and N treatment), with a shape parameter of 1 (weak, to account for phylogenetic uncertainty), and using 1,000,000 iterations with a sample interval of 50 and a burn‐in of 100,000. GLMM trace plots were free of pattern, and autocorrelation among samples calculated between iterations were all <0.1, indicating that the model had performed well (Hadfield 2010).

## Results

**Figure 1:**
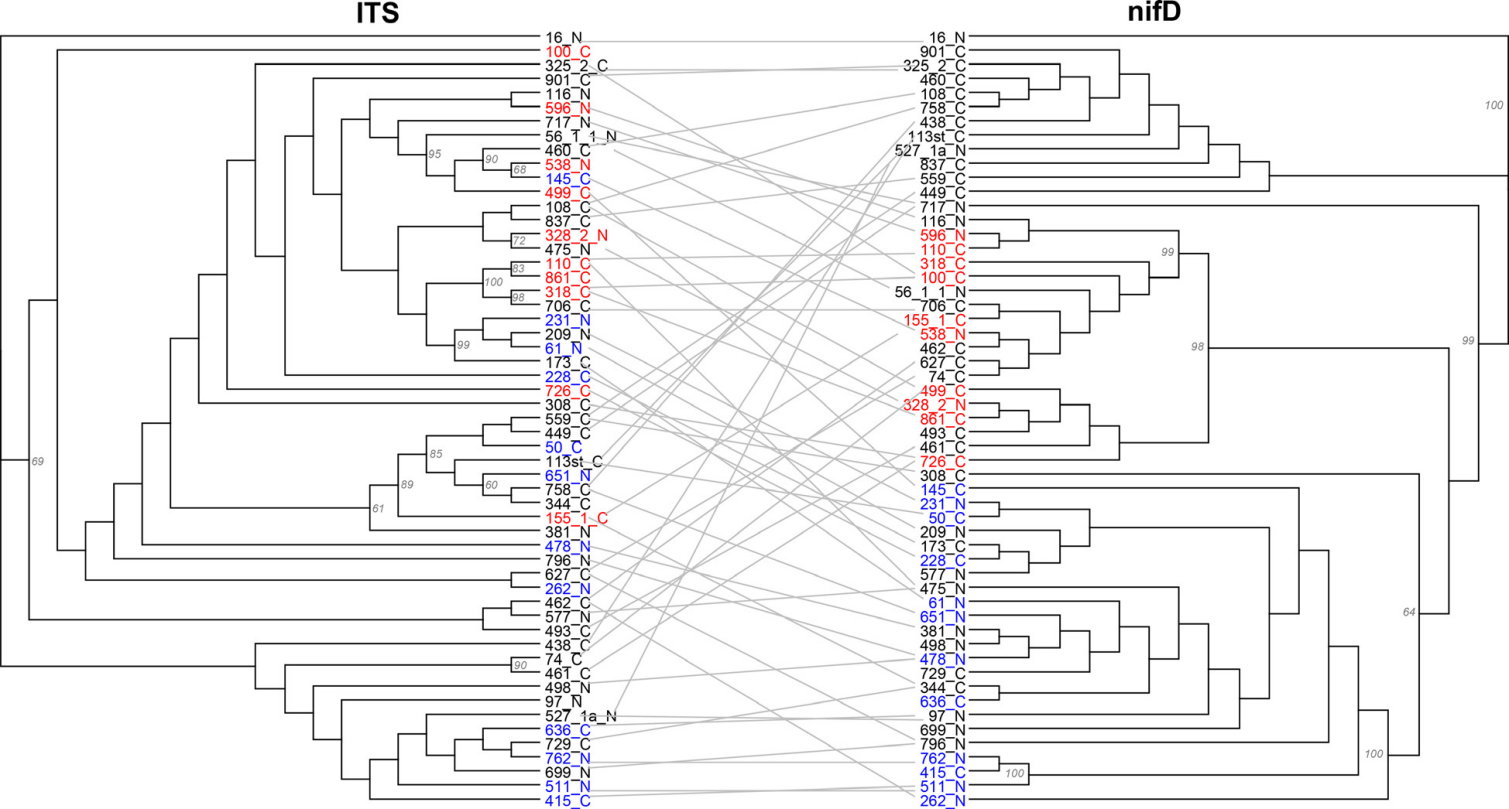
Tanglegram, ITS/nifD – A tanglegram highlighting patterns of phylogenetic variation between the ITS and nifD phylogenies. All nodes with bootstrap support ≥60 are labeled on the phylogenies. Strains in blue represent poor partners and strains in red represent superior partners visually identified from trait mapping (see Fig. 3).

The SH test rejected the null hypothesis of congruence for all pairwise comparisons of loci in our study (Table 1), consistent with some amount of recombination among all loci; the PACo test, however, rejected the null hypothesis of random association and instead supported significant topological congruence in comparisons among all genes (Table 1). Comparisons of the ITS and pSym genes resulted in less congruence via PACo and a high degree of entanglement in tanglegrams compared to among‐pSym comparisons (Table 1; Figs. 1, S1–5). When comparing among just the three pSym loci, *nifH* and *nodD‐A* were most congruent (Table 1; Fig. S4), while and *nifD* and *nifH* were least congruent (Table 1; Fig. S3). In total, these analyses indicate some amount of phylogenetic conflict among loci, but do suggest that the pSym loci are more genealogically concordant with each other compared to the ITS. While the SH test was significant for all comparisons, it is important to note that this test is well known to be conservative (Buckley 2002). Moreover, both of these analyses do not account for phylogenetic uncertainty, which is apparent in these datasets by the overall low bootstrap supports. The pSym datasets are particularly information‐limited due to low levels of phylogenetically informative characters (*nifD*: 16/456; *nifH*: 19/834; *nodD‐A*: 42/447) relative to the ITS (114/807) and incomplete taxon sampling due to PCR failure despite repeated attempts (≥3 attempts/strain/locus) (*nifD*: 55/63; *nifH*: 54/63; *nodD‐A*: 37/63). PCR failure might be due to sequence divergence, gene rearrangement, or gene loss, potentially of the entire plasmid.

**Table 1:** Above the diagonal: Shimodaira–Hasegawa test (SH test: ln L diff). Below the diagonal: PACo (Procrustean Approach to Cophylogeny: m^2^ = lack‐of‐fit)

Locus	*ITS*	*nifD*	*nifH*	*nodD*
*ITS*		1551***	1531***	793***
*nifD*	0.824****		1162*	106***
*nifH*	0.921***	0.161****		66**
*nodD*	0.541***	0.114****	0.090****	

****< 0.0001; ***< 0.001; **< 0.01; *< 0.05.

**Figure 2:**
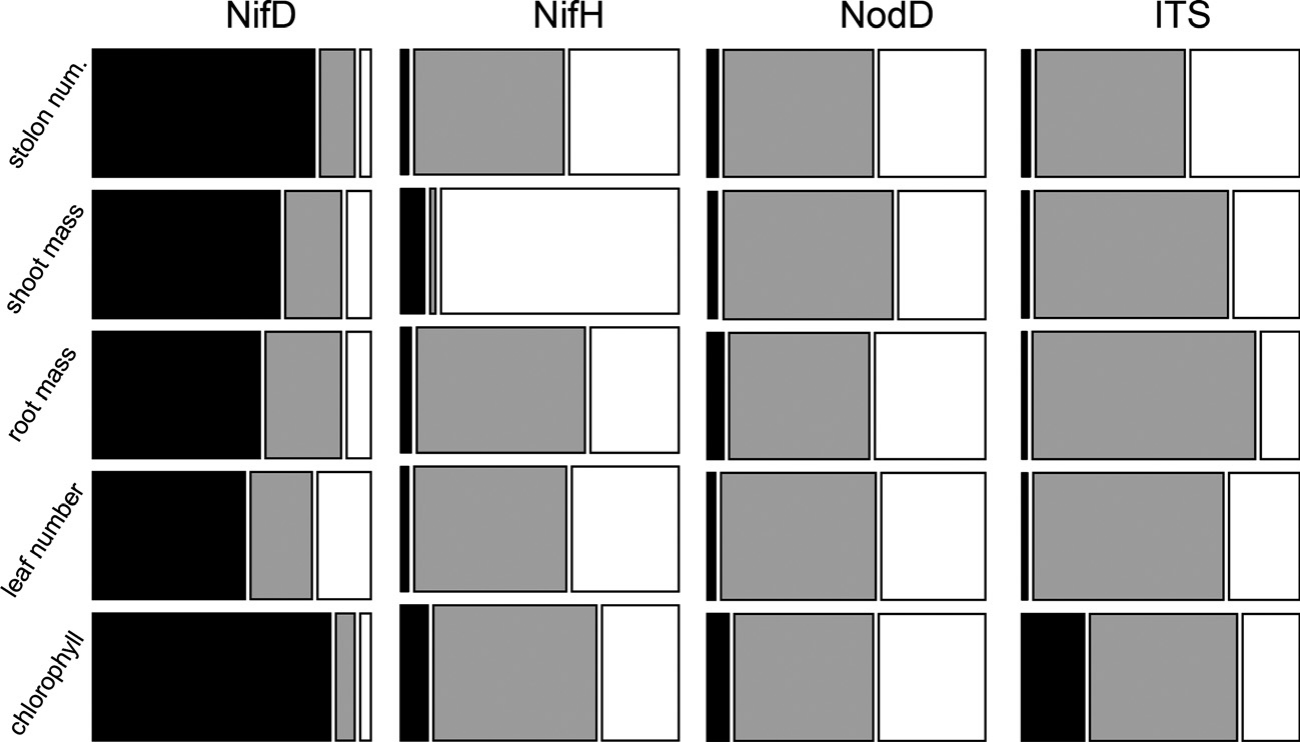
Mosaic plot, GLMM attributable variance – The percent of variance attributable to phylogenetic relationships versus N fertilization treatment. Black: variation attributable to phylogeny; gray: variation attributable to N fertilization; white: residual variance.

**Figure 3:**
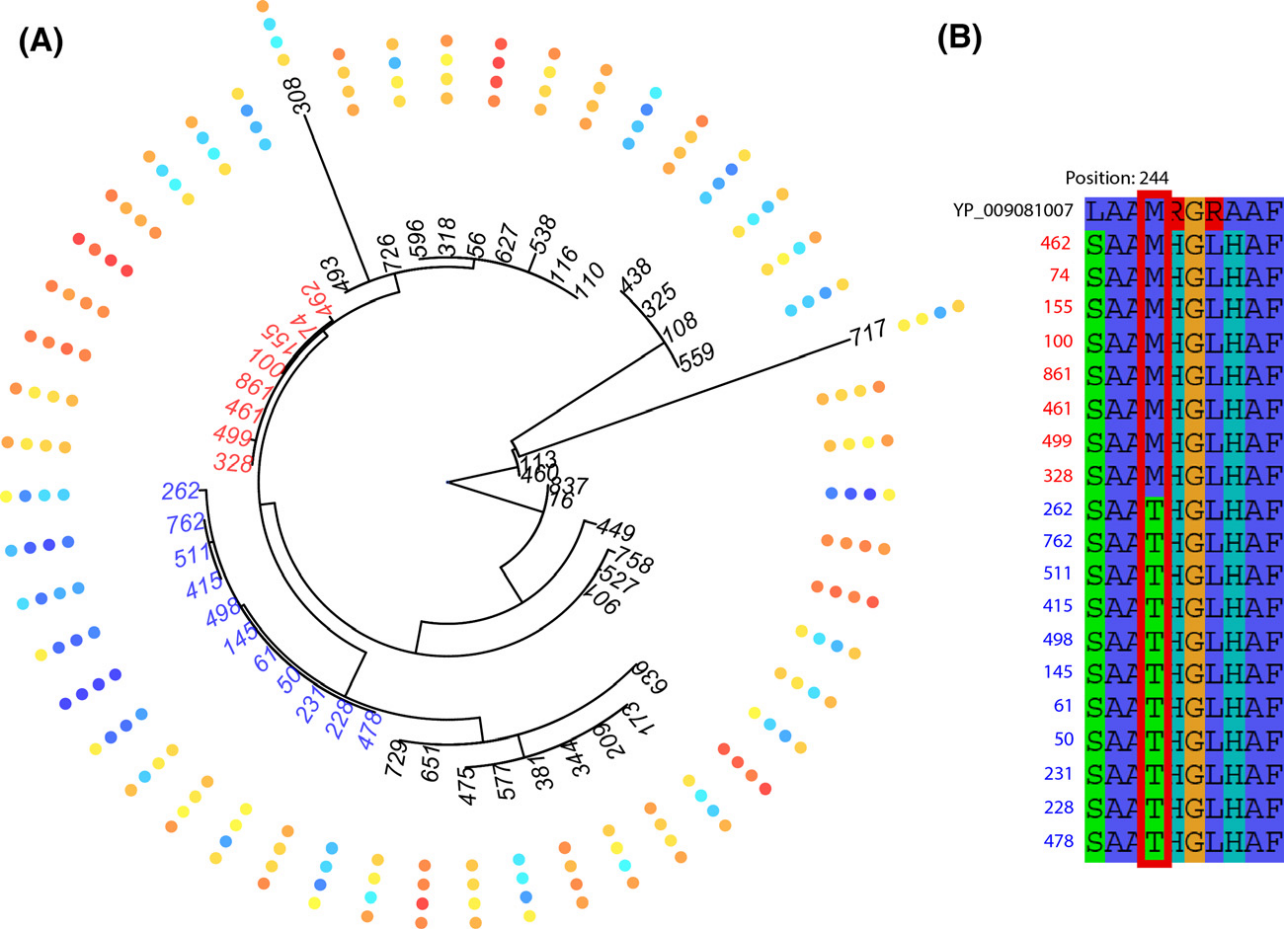
Bullseye plot, nifD/Partner Quality – (A) Fan phylogeny of nifD with measure of partner quality mapped to the tips. Traits, from inside out: shoot mass, leaf number, stolon number, and chlorophyll content. Darker colors represent values that are further away from the mean, with blue indicating below the mean (−) and red indicating above the mean (+). Strains from two clades are highlighted: the clade in blue is predominantly below the mean, while the clade in red is predominantly above the mean. (B) Amino acid alignment of residues 241–250 of nifD, with a MoFe subunit interaction site (residue 244) outlined in red; the same strains are highlighted as above.

We found that partitioning of partner quality variation depended considerably on the locus analyzed. The N fertilization treatment explained the majority of rhizobium partner quality for nearly all models that included the ITS, *nifH,* and *nodD‐A* phylogenies (Fig. 2). These findings would suggest that loci elsewhere in the genome (i.e., apart from these three marker loci) have changed in response to N fertilization. Conversely, in our analysis of *nifD*, phylogeny explained the vast majority of partner quality variation (Fig. 2), and two clades of generally high‐ and low‐quality stand out when partner quality is mapped to the tips of the tree (Fig. 3A), potentially implicating genetic changes within *nifD* in partner quality variation. Bullseye plots showing partner quality mapped to *ITS*,* nifH*, and *nodD‐A* can be found in Figure S6. In contrast to the other partner quality traits (leaf number, stolon number, and plant biomass), the ITS phylogeny accounted for a large proportion (23.4%) of the variance in chlorophyll content (Fig. 2); moreover, including N treatment in the model of chlorophyll content did not improve model fit. These results suggest that causative loci underlying variation in partner chlorophyll content might be linked to the ITS region, most likely somewhere on the chromosome.

Using an amino acid alignment of *nifD* with the reference, we determined that the reference along with all of the strains from the high‐quality clade have a methionine at residue 244, whereas all of the strains from the low‐quality clade possess a tyrosine (Fig. 3B). Residue 244 is a MoFe subunit interaction site, and previous studies have shown that changes to these sites can alter a bacterium's ability to fix N (Brigle et al. 1987). Additionally strain 116, which stands out as a low‐quality member of a high‐quality subclade, is the only strain in the clade that possesses a mutation at residue 100, another MoFe subunit interaction site.

## Discussion

Mutualisms are thought to be susceptible to anthropogenic change, although the ecological and evolutionary processes that drive mutualism decline are not yet understood. Microbes are key players in many important mutualisms, including the N‐fixing legume–rhizobium symbiosis; therefore, understanding the evolution of rhizobium partner quality in natural populations can shed light on the evolution of bacteria in nature and the maintenance of mutualisms in general. Building on our previous phenotypic analysis (Weese et al. 2015), which indicated that rhizobia from N‐fertilized plots were less‐beneficial symbionts of clover, we compared evolutionary histories of four gene sequences (one on the chromosome and three pSym genes) with phylogenetic mixed modeling to investigate the evolutionary process underlying the observed reductions in partner quality. Our results indicate that the ITS region and the pSym have different evolutionary histories and relationships with rhizobium partner quality, but that patterns for chlorophyll content differed from other measures of partner quality. Our results differ from expectations under the ecotype model with zero recombination, but do suggest significant phylogenetic signal in partner quality, particularly at *nifD*, but also the ITS for chlorophyll content. We discuss these main results below.

Our findings indicate both phylogenetic signal and N treatment contribute to among‐strain variation in partner quality and thus implicate evolutionary changes in response to experimental N treatments as well as among‐lineage variation for mutualism. Given the results of our quantitative trait modeling and various analyses of congruence/coevolution, however, it appears that the pSym and the chromosome have different evolutionary histories. By contrast, more congruence among the three pSym genes confirms that these genes are in partial linkage and that they are more likely to move as a functional unit (Broughton et al. 1984). An alternative explanation is that phylogenetic uncertainty is driving incongruence, as co‐diversifying genes that lack in phylogenetic signal may appear incongruent due to random noise (Zaneveld et al. 2008). Given previous work showing that rhizobium symbiosis plasmids are self‐transmissible (Bittinger et al. 2000) and that novel symbiosis islands can confer a strong selective advantage (Parker 2012; Horn et al. 2014), it is possible that recombination or HGT of the pSym across ITS backgrounds has led to the differing signals in the various loci.

What do our data suggest about the evolutionary process leading to partner quality decline in high N plots? Because we find evidence for both phylogenetic signal at our marker loci (particularly at *nifD*) and unlinked evolutionary changes in response to N treatment, we can reject a strict one‐to‐one relationship between phylogeny and partner quality and thus strict clonal evolution. Nevertheless, the correct interpretation of these findings regarding the evolutionary process that has occurred during the last 22 years depends somewhat on the age of these rhizobium lineages, compared to the length of the ecological experiment. On the one hand, if the phylogenetic signal in our dataset predates the initiation of LTER treatments (1988), this would suggest a combination of lineage sorting (changes in abundance of pre‐existing lineages) and microevolutionary changes at causative genes in driving the difference in partner quality between N‐evolved and control plots. On the other hand, if these lineages have diversified because the N treatments were first established, then our findings would suggest that particular lineages have increased in frequency along with adaptive changes in partner quality. Another interpretation would be that 22 years is not long enough for complete lineage sorting of recent substitutions. Given current best estimates of point mutation rates in bacteria (Wielgoss et al. 2011), we would expect approximately four mutations over the course of our 22‐year experiment, yet we find 19 informative sites, which implies that the phylogenetic signal predates the LTER treatment. Nevertheless, all three scenarios imply a mix of clonality and recombination consistent with other recent studies of bacteria in nature (Feil et al. 2001; Hanage et al. 2006; Narra and Ochman 2006; Pérez‐Losada et al. 2006; Vos and Didelot 2009).

Our results showing that the *nifD* phylogeny accounts for the vast majority of variation in partner quality might suggest a causal relationship between the locus and measures of partner quality, particularly given the lack of variation accounted for by phylogeny at *nifH* or *nodD‐A*. This would be consistent with the known importance of *nifD* in the mutualism (Hennecke 1990; Spaink et al. 1998), as well as a previous study showing that mutations at the MoFe subunit interaction sites can lead to changes in the efficiency of N fixation, resulting in a range of effects on growth in N‐limiting conditions despite normal accumulation levels of both MoFe subunits (Brigle et al. 1987).

While we are unable to rule out the possibility of linked loci elsewhere on the plasmid, it should be noted that there is divergent signal between *nifD* versus the structurally adjacent *nifH*/*nodD‐A* despite evidence of co‐diversification, which implies a lack of causally linked loci near our markers. The contribution of chromosomal loci is inconclusive based on sequencing the ITS alone. Understanding which loci determine partner quality and underlie the difference between N‐evolved and control rhizobia will require genomewide sequencing, which is currently ongoing. Our current evidence suggests that variation in *nifD* might have played a role in contributing to the reduction in partner quality in N‐fertilized strains. The mechanism leading to a change in allele frequency remains unclear; nevertheless, our data are compatible with conjugative transfer of the pSym and subsequent homologous recombination of the *nifD* locus, a hypothesis consistent with previous findings on the architecture and nature of rhizobial plasmids (Mercado‐Blanco and Toro 1996; Herrera‐Cervera et al. 1998; Ding et al. 2013).

Interestingly, the ITS phylogeny accounted for considerable variance in chlorophyll content (>23.4%), a key measure of partner quality; this was quite different from other measures of partner quality, where phylogeny never accounted for >3.2% of variation in partner quality. This finding seemingly contrasts with the fact that chlorophyll content and other measures of partner quality are correlated among strain means (chlorophyll content ~ shoot mass, *r* ^2^ = 0.87, *P* < 0.0001; chlorophyll content ~ leaf number, *r* ^2^ = 0.80 *P* < 0.0001). Partner quality traits are quantitative, varying more‐or‐less continuously among strains (Weese et al. 2015); therefore, these complex phenotypes are likely to be underlain by multiple loci, some of which might contribute more to some traits than others. Our findings might suggest that the ITS region is in partial linkage with loci that are important in determining plant chlorophyll content, but more work would be needed to test this hypothesis.

## Conclusion

Here, we have found that phylogenetic variation at *nifD* explains much of the variation in partner quality, and we suggest MoFe subunit interaction sites as candidates in the reduction in rhizobium partner quality. We find that the phylogenies of pSym genes are not consistent with the phylogeny of the ITS region, but are broadly similar to each other. These results allow for a number of evolutionary scenarios and further sequencing will be required to determine whether additional changes throughout the genome have driven reductions in partner quality, as well as to elucidate the role of recombination and HGT on whole genomes.

## Data Archival Location

Genbank (sequence) and TreeBASE (phylogenies).

## Conflict of Interest

The authors declare no conflict of interest.

## Supporting information


**Figure S1.** Tanglegram of ITS/nifH.
**Figure S2.** Tanglegram of ITS/nodD.
**Figure S3.** Tanglegram of nifD/nifH.
**Figure S4.** Tanglegram of nifD/nodD.
**Figure S5.** Tanglegram of nifH/nodD.

**Figure S6.** Bullseye plot showing partner quality mapped to the fan phylogenies of *ITS*,* nifH*, and *nodD‐A*. Traits, from inside out: shoot mass, leaf number, stolon number, and chlorophyll content. Darker colors represent values that are further away from the mean, with blue indicating below the mean (‐) and red indicating above the mean (+).
