# Supplementary figures and images for: Decoupled genomic elements and the evolution of partner quality in nitrogen‐fixing rhizobia
Source: Ecol Evol. 2016 Jan 28;6(5):1317–27. doi: 10.1002/ece3.1953 (PMC4775534; doi:10.1002/ece3.1953)

Figure S2

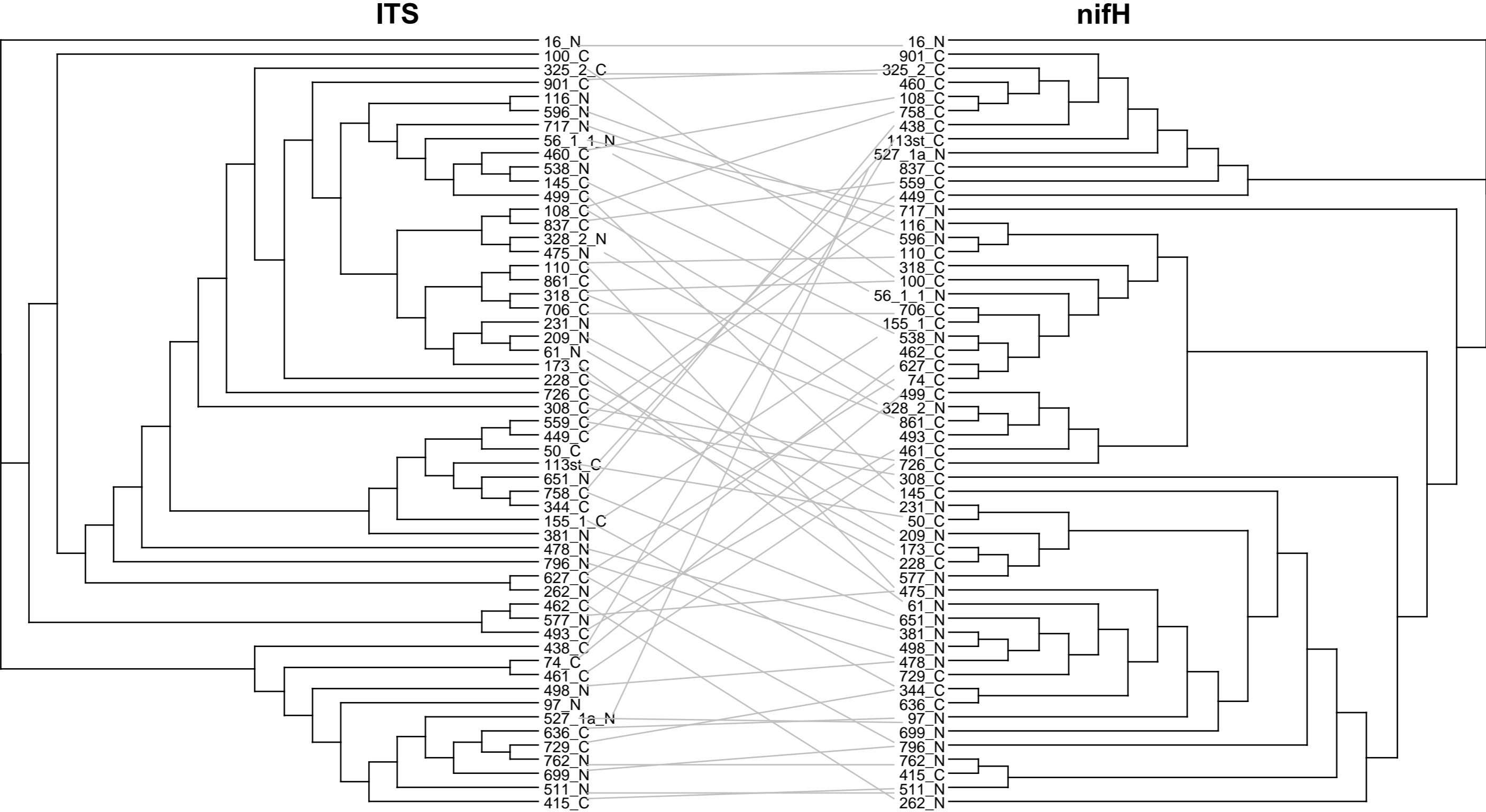

Figure S3

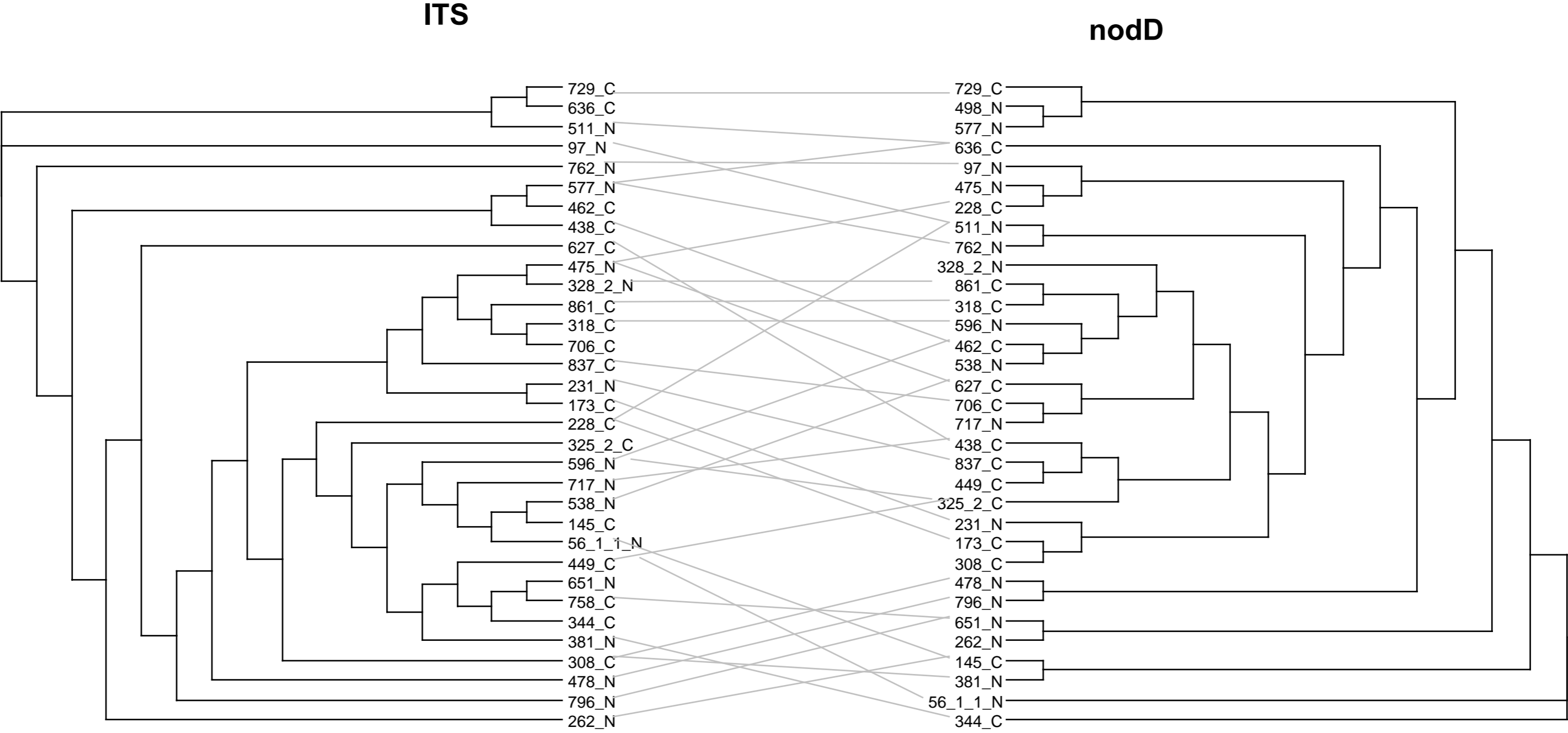

Figure S4

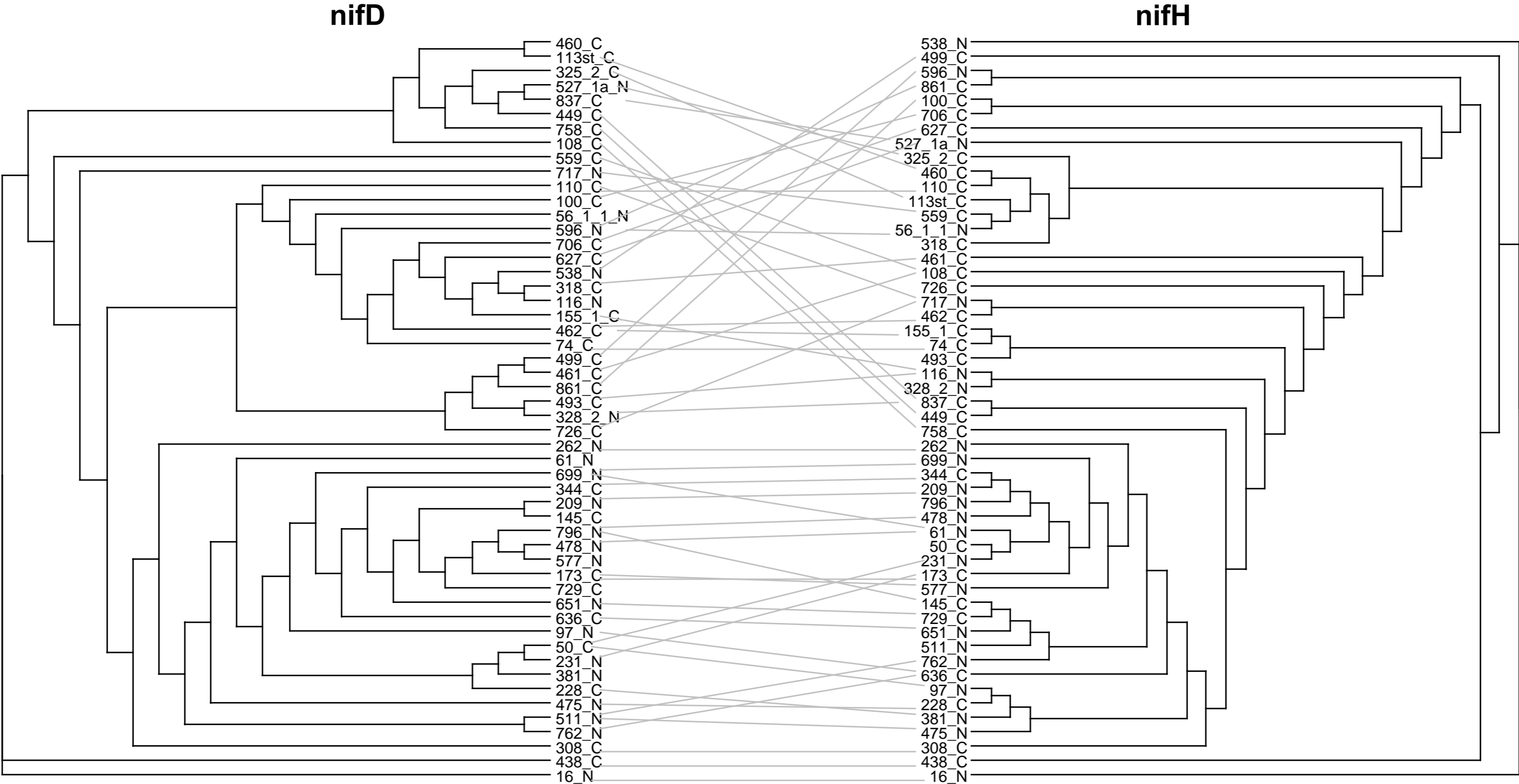

Figure S5

nifD

nodD

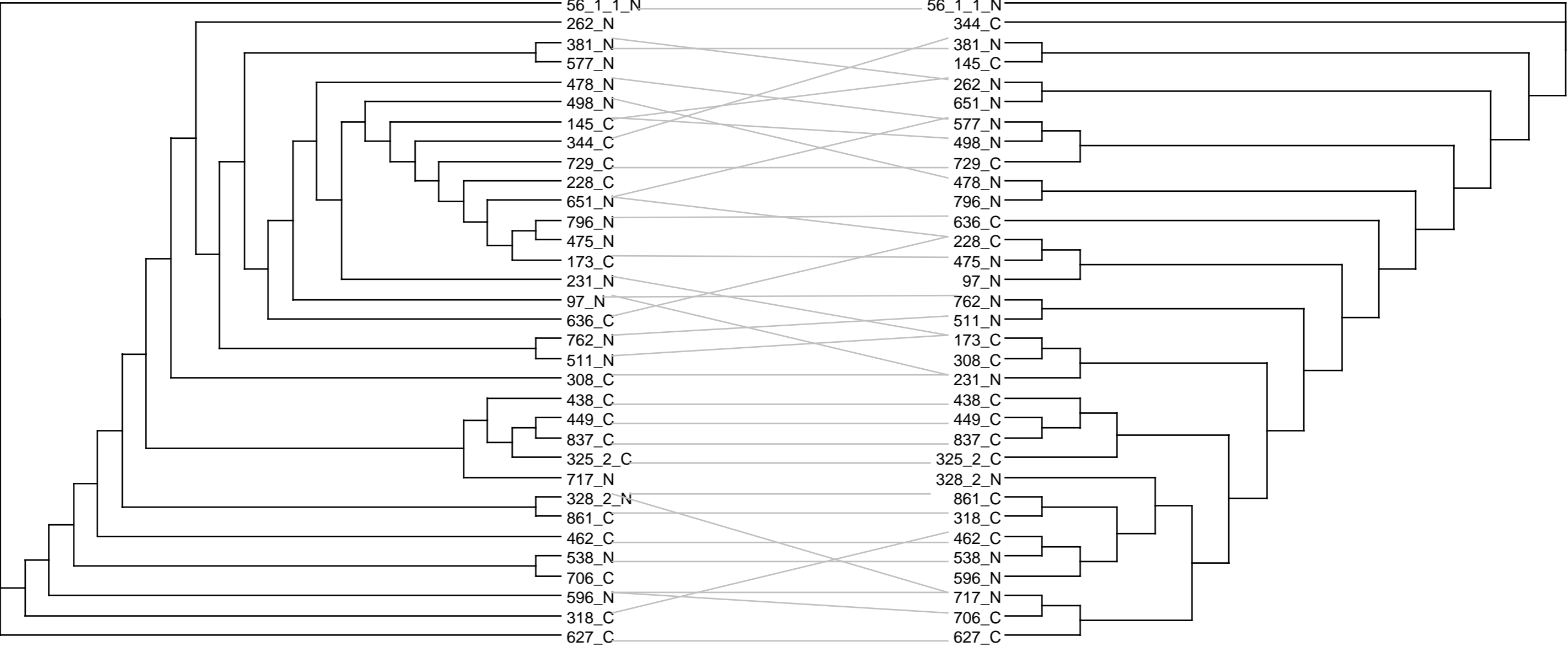

Figure S6

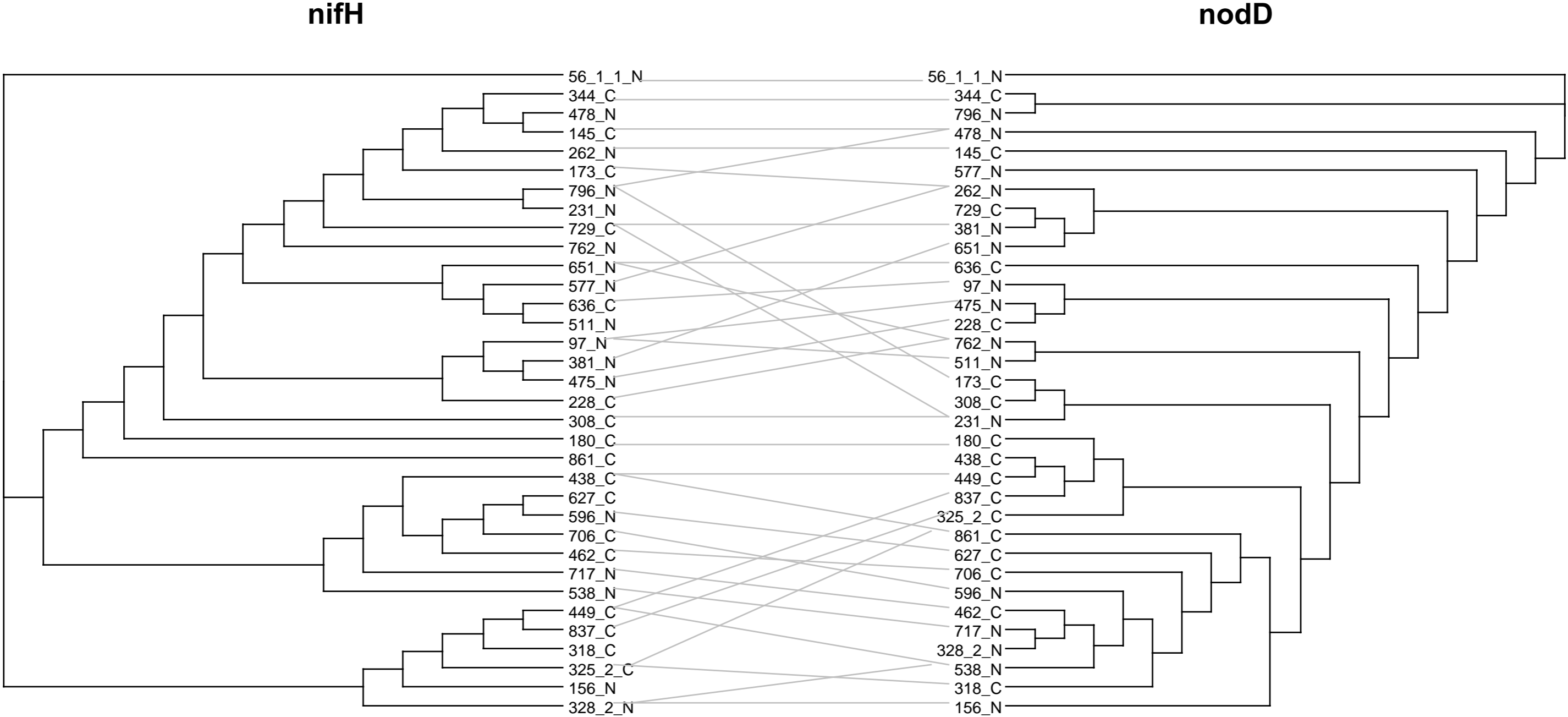

Supplement: Supplementary file 1 — Figure S1. Tanglegram of ITS/nifH. Figure S2. Tanglegram of ITS/nodD. Figure S3. Tanglegram of nifD/nifH. Figure S4. Tanglegram of nifD/nodD. Figure S5. Tanglegram of nifH/nodD. [file ECE3-6-1317-s001.pdf]

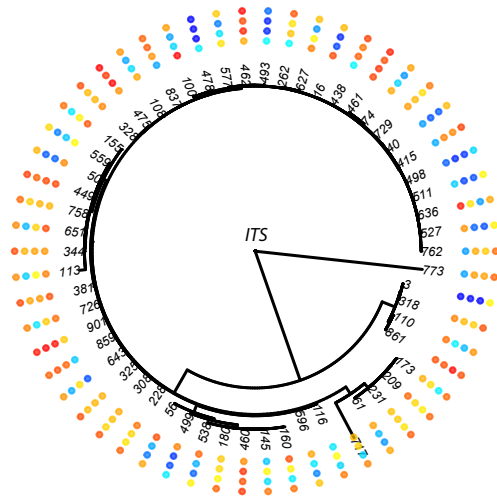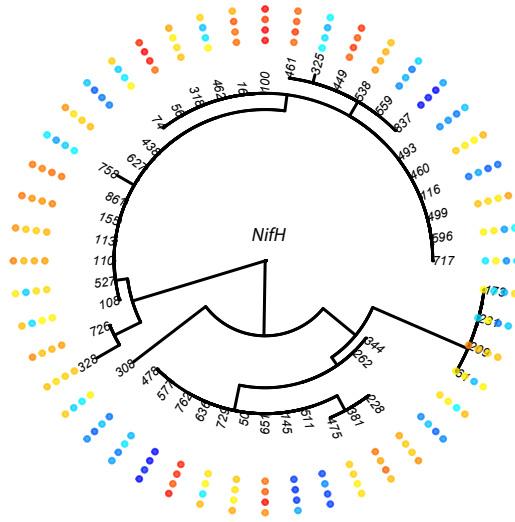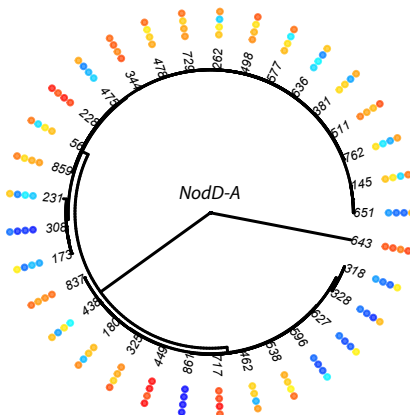

Supplement: Supplementary file 2 — Figure S6. Bullseye plot showing partner quality mapped to the fan phylogenies of ITS, nifH, and nodD‐A. Traits, from inside out: shoot mass, leaf number, stolon number, and chlorophyll content. Darker colors represent values that are further away from the mean, with blue indicating below the mean (‐) and red indicating above the mean (+). [file ECE3-6-1317-s002.pdf]
